# Supportive and palliative care indicators tool (SPICT™): content validity, feasibility and pre-test of the Italian version

**DOI:** 10.1186/s12904-020-00584-3

**Published:** 2020-06-06

**Authors:** Giuseppe Casale, Caterina Magnani, Renato Fanelli, Laura Surdo, Mauro Goletti, Kirsty Boyd, Daniela D’Angelo, Chiara Mastroianni, the SPICT-IT™ study group, Maurizio Cancian, Marco Colotto, Antonella Di Giacomo, Giuseppe Fucito, Giorgio Gentili, Patrizia Latorre, Pierangelo Lora Aprile, Michele Massaro, Andrea Pace, Antonella Savarese, Simone Scarlata, Maria Consiglia Stefanelli, Adriana Turriziani

**Affiliations:** 1 Antea Associazione, Piazza Santa Maria della Pietà, 5, Pad XXII, 00135 Rome, Italy; 2 Roma 1 Local Health Authority, Borgo Santo Spirito, 3, 00193 Rome, Italy; 3 Primary Care Medical Center, Via Frà Albenzio, 10, 00136 Rome, Italy; 4 Primary Palliative Care Group, The University of Edinburgh, Doorway 3, Medical School, Teviot Place, Edinburgh, EH8 9AG UK; 5 Research, National Institute of Health, Via Giano della Bella, 34, 00162 Rome, Italy

**Keywords:** Palliative care, SPICT, Italian, Supportive care, Primary care

## Abstract

**Background:**

Difficulties in identifying patients at risk of clinical deterioration or death represent one of the main barriers to Palliative Care (PC) development in the community. Currently, no specific Italian tools aimed at identifying patients with PC needs are available. Of the different European tools available, the SPICT™ can be used easily in any kind of setting and does not include the Surprise Question. The purpose of the study was to translate, cross-culturally adapt and pre-test the Italian version of the SPICT™.

**Methods:**

The Beaton recommendations for the cross-cultural adaptation of instruments were followed. Content validity was assessed using the Lynn method. A sample of Italian General Practitioners (GPs) assessed the SPICT-IT™ for feasibility and tested it.

**Results:**

During the cross-cultural adaptation, some issues regarding semantic, experiential, idiomatic and conceptual equivalences were raised and resolved. The Scale-Content Validity Index/Ave was 0.86. Of the 907 GPs included in the sample, 71 (7.8%) agreed to test the SPICT-IT™ and to assess its feasibility. The participants provided care for 73,526 people in the community. Of these people, 1.7% (*N* = 1303) were identified as being in need of PC according to the SPICT-IT™. Sixty-six (93.0%) GPs stated they would use the SPICT-IT™ in their daily clinical practice.

**Conclusions:**

The SPICT-IT™ demonstrated acceptable content validity. The percentage of patients identified through the SPICT-IT™ was comparable to findings from literature. The next phase of this project will investigate the impact of a proactive training programme aimed at supporting GPs in identifying patients with PC needs and delivering appropriate Primary Palliative Care (PPC).

## Background

Difficulties in identifying patients at risk of clinical deterioration or death have been recognised as one of the main barriers for palliative care (PC) development and integration in the community [1]. European countries have been encouraged by the World Health Organization (WHO) to develop research initiatives that aim to overcome the barriers to Primary Palliative Care (PPC) development [1, 2].

Identification of those individuals who may benefit from palliative care early in their illness trajectory should be the first step in PPC development. Patients identified as at risk of deteriorating or of dying, will still require assessment of symptoms and needs across all domains to determine whether they would benefit from PC, or if they have unmet PC needs. Through identification and assessment GPs will be able to plan and manage the most appropriate care for patients [3].

Effective end-of-life-care planning must begin with the identification of patients at risk of dying. Recent literature reports that several diagnostic [4] symptoms and needs assessment tools [5] are available. Assessment tools or patient-report outcome measures are often used, especially in cancer patients [6]. The available diagnostic or screening tools (used by clinicians) for the identification of patients at risk of deterioration or death, although very useful for helping general practitioners (GPs), are rarely used in clinical practice [7].

The Supportive and Palliative Care Indicators Tool (SPICT™) (see Additional file 1) is one of those tools. It is easy to use, complete and supports clinicians in identifying unmet needs through actual care evaluation and the analysis of signs of deterioration in adult patients (age > 18); its use is approved for both primary care and acute hospitals [8]. The SPICT™ does not include the Surprise Question (SQ) and through its use, the risk of “prognostic paralysis” (associated with predicting life expectancy), which may result in the delayed identification of people with PC needs, is avoided [9]. Patients with PC needs could have a prognosis of over 1 year, especially where conditions other than cancer are concerned. The SPICT™ was originally developed in 2010 by the Primary Palliative Care Research Group of the University of Edinburgh to help clinicians identify patients with advanced conditions who might benefit from primary or, in some cases, specialised PC [8]. It comprises a set of clinical indicators and has a three-part structure. The first section consists of general clinical indicators, the second details specific illness indicators and the third provides essential recommendations for reviewing and planning care [8, 10]. Physicians working in any kind of care setting can screen patients using the checklist of indicators included in the SPICT™. People who have been identified as needing PC by means of the SPICT™ usually have at least two general indicators [10, 11]. The SPICT™ has been translated and culturally adapted in different languages, but not Italian [10, 12]. This absence could limit both national development of PC and comparisons with other European countries. Therefore, the main aim of this study was to translate, culturally adapt and assess the content validity and feasibility of the SPICT-IT™. The secondary aims were to test the SPICT-IT™ with the help of a sample of general practitioners (GPs) and to assess how frequently the tools for identification of PC needs are used in real-life scenarios.

## Methods

Lazio 1 Ethics Committee (Rome, Italy) approved the study. Permission to translate the SPICT™ was provided by the Edinburgh Primary Palliative Care Research Group.

### Translation and cross-cultural adaptation

The Italian translation and cross-cultural adaptation of the SPICT™ was carried out according to the Beaton protocol [13] and the WHO recommendations for translation and adaptation of instruments [14].

Stage 1: Two native Italian speakers translated the tool into Italian, with the translations done in parallel, but independently.

Stage 2: The two translators synthesised the results of the translations. A written report was produced to document the synthesis process.

Stage 3: A translator who did not have a medical background and whose native language was English performed the back translation. Agreement between the translated version and the original version was verified to ensure that the content of the translated version matched that of the original.

Stage 4: A panel of 10 experts reviewed the translated version to reach a consensus on any discrepancies. The expert committee was composed of: 1 methodology researcher, 1 physician with an advanced degree in internal medicine, 2 GPs, 2 PC physicians, 1 oncologist, 1 nurse researcher, 1 PC nurse and the two native Italian translators. Any issues regarding semantic, idiomatic, experiential or conceptual equivalences were discussed and resolved. Subsequently, the pre-final version of the SPICT-IT™ was submitted to a panel of 11 experts (different from the 10 experts involved in the first panel) for assessment of the SPICT-IT™ content validity [15, 16]. The panel consisted of 5 GPs, 1 neurologist, 1 oncologist, 1 geriatrician, 1 respiratory medicine specialist, 1 cardiologist and 1 gastroenterologist; each of these individuals had documented experience in delivering PC. The experts received an anonymised electronic questionnaire in which they were asked to rate each of the clinical indicators, in terms of clarity and relevancy, on a four-point Likert scale (not relevant = 1; somewhat relevant = 2; quite relevant = 3; highly relevant = 4). Finally, an electronic questionnaire, together with the SPICT-IT™, was distributed to all GPs (*N* = 907) in the Roma 1 Local Health Authority for feasibility assessment and testing. Each healthcare district director encouraged the GPs to take part in this project by sending a letter that explained the importance of developing PPC in Italy. Each GP was asked to provide the total number of patients (age > 18) in care at the time of submitting the questionnaire. Furthermore, they were asked to identify how many of these patients (age > 18) had at least 2 general indicators and 1 specific indicator from among those included in the SPICT-IT™.

### Statistical analysis

Quantitative data were analysed with descriptive statistics. If they were related to the quantitative variables, data were summarised by using means, standard deviations, medians and ranges. The Content Validity Index for Item (CVI-I) was calculated as the relationship between the number of experts on the panel who rated the indicators 3–4 and the total number of experts for each of the clinical indicators. The Content Validity Index for Scale (CVI-S) was calculated by using the S-CVI/Average as included in the Lynn method (an S-CVI/Ave >.80 is the standard criterion for acceptability for the S-CVI) [15]. The multi-rater kappa statistic, with an adjustment for a chance agreement, was calculated to supplement the I-CVI because the kappa statistic provides information concerning the degree of agreement beyond chance (kappa values are considered excellent when above 0.74, good between 0.60 and 0.74, and fair between 0.40 and 0.59) [16].

## Results

During the cross-cultural adaptation of the SPICT™, researchers identified the following main discrepancies with respect to the four types of equivalences:

### Semantic equivalence

- The experts agreed to add “mechanical, invasive or non-invasive ventilation” to the item known as “has needed ventilation”, to specify that non-invasive ventilation also had to be considered.
- The expression “plan care” was not literally translated because the experts believed that it may result in healthcare professionals (HCPs) considering only “medical care”.

### Conceptual equivalence

- The experts agreed that the literal translation of “too frail for cancer treatment” was not easy to understand in Italian; therefore, it was translated as “general status that does not allow starting or continuing specific cancer treatment”.

### Idiomatic equivalence

- The expression “life-limiting conditions” was not literally translated. The panel decided to translate it as “conditions with a limited prognosis”.

### Experiential equivalence

- The word “unmet” that was used before “PC needs” may result in HCPs underestimating the number of patients to be identified. The panel agreed to omit the word “unmet” in the Italian version to avoid this potential problem.

The multi-rater kappa statistic, which was adjusted for a chance agreement, was > 0.74 for all items. The S-CVI/Ave was 0.86.

Of the 907 GPs invited to take part in the study, 71 (7.8%) GPs agreed to participate and responded to the questionnaire. Males comprised 61% of participating GPs, and the mean age was 58.3 ± 8.7 (Table 1). The mean time for reading and applying the SPICT-IT™ was 8.5 ± 5.3 min. 97.2% (*N* = 69) of GPs reported that the SPICT-IT™ was clear and comprehensible. Two GPs reported that the SPICT-IT™ included too many indicators, and that it was repetitive and too complex. The GPs who participated in the study provided care for 73,526 people (Table 1) in total. Of these, 1303 patients had at least 2 general indicators and 1 specific indicator for illnesses. A mean of 1.7% of individuals was therefore identified as requiring PC (Table 1). 94.4% (*N* = 67) of the GPs stated that they never use any tools for identifying PC needs. Four GPs reported that clinical evidence is sufficient for identifying patients with PC needs and that “good sense is the best tool”. However, 93.0% (*N* = 66) of the GPs stated that they would use the SPICT-IT™ in daily clinical practice.

**Table 1:** GPs characteristics

GPs characteristics	***N*** = 71 (%)
**Age, mean (SD)**	58,3 (+ − 8,7)
**Gender**	
Male	43 (61%)
Female	28 (39%)
**Years of experience as GP**	
< =5	5 (7%)
5–15	6 (8%)
15–25	12 (17%)
> =25	48 (68%)
**Total No. of patients cared for**	73,526 (100%)
Total No. of patients with PC needs*	1303 (1.7%)

*No. of patients cared for by the GPs with at least 2 general indicators and 1 specific of those included in the SPICT™

## Discussion

Demographic changes across Europe, such as an aging population and the rise of chronic degenerative diseases, highlight the increasing numbers of people with PC needs [17].

In 2014 the WHO Global Atlas of PC at the End of Life clarified the inclusive nature of PC, stating that PC should also be delivered to patients who are affected by chronic conditions, that it should be provided in any setting on the basis of need and not based on diagnosis or prognosis [18]. Consequently, accurate identification of people with PC needs is essential for the development of PC at all levels. Furthermore, early PC interventions proved effective in improving quality of life [19, 20]. In this context, the research presented here is an essential initiative that aims to contribute to PC development. During the translation and cultural adaptation process, minimal linguistic or cultural discrepancies were encountered. Generally, a good level of content validity was observed in the SPICT-IT™. Our group of GPs’ average time for applying the SPICT-IT™ was shorter than the reported time for application of the Spanish translation [11]. We believe that use of the SPICT-IT™ as part of routine care in real-life clinical contexts would reduce the time required even further as clinicians grew familiar with it. In this study, 7.8% of the GPs approached agreed to participate. This confirms the difficulties involved in recruiting GPs for PC-focussed projects. Data from a recent study reported that of 4065 eligible GPs, only 2.8% agreed to participate in a PPC research in Belgium [21]. Tailored training programmes should be implemented to encourage more GPs to take part in this type of research.

The prevalence of patients who were identified through the SPICT-IT™ (1.7%) was similar to that reported in a recent Italian multicentre study [22].

## Study limitations

This study has some limitations.

First of all, the Beaton method is commonly used for cross-cultural adaptation of self-report measures, while the SPICT™ is a set of clinical indicators. Secondly, the low response rate from GPs will require further interventions to raise GPs’ awareness on the importance of promoting PC research. Finally, the validity of the SPICT-IT™ should be further assessed by registering and monitoring data of identified patients.

## Conclusion

The Italian version of the SPICT™ exhibited acceptable feasibility and content-validity. The prevalence of patients in need of PC who were identified through the SPICT-IT™ was similar to that reported in literature. The next phase of this project is under way, and it will provide information for interactive educational programmes concerning the use of PPC for GPs and hospital physicians. Alongside the better use of effective tools to identify people with palliative care needs, there should be development and dissemination of these tools to help clinicians assess the nature and complexity of PC needs and deliver the most appropriate care for this population.

## Supplementary information

**Additional file 1.**

## Data Availability

The datasets used and/or analysed during the current study are available from the corresponding author on reasonable request.

## Abbreviations

GP: General Practitioner
PC: Palliative Care
WHO: World Health Organization
PPC: Primary Palliative Care
SPICT: Supportive and Palliative Care Indicators Tool
CVI-I: Content Validity Index for Item
CVI-S: Content Validity Index for Scale
HCP: Healthcare professional

## References

[1] Murray SA, Firth A, Schneider N (2015). Promoting palliative care in the community: production of the primary palliative care toolkit by the European Association of Palliative Care Taskforce in primary palliative care. Palliat Med.

[2] World Health Organization (2014). Strengthening of palliative care as a component of integrated treatment throughout the life course. J Pain Palliat Care Pharmacother.

[3] Clifford C, Thomas K, Armstrong Wilson J (2016). Going for Gold: the Gold Standards Framework programme and accreditation in primary care. End Life J.

[4] Walsh RI, Mitchell G, Francis L, van Driel ML (2015). What diagnostic tools exist for the early identification of palliative care patients in general practice? A systematic review. J Palliat Care.

[5] Bausewein C, Daveson BA, Currow (2016). EAPC white paper on outcome measurement in palliative care: improving practice, attaining outcomes and delivering quality services–recommendations from the European Association for Palliative Care (EAPC) task force on outcome measurement. Palliat Med.

[6] Collins ES, Witt J, Bausewein C (2015). A systematic review of the use of the palliative care outcome scale and the support team assessment schedule in palliative care. J Pain Symptom Manage.

[7] Maas EAT, Murray SA, Engels Y (2013). What tools are available to identify patients with palliative care needs in primary care: a systematic literature review and survey of European practice. BMJ Support Palliat Care.

[8] Highet G, Crawford D, Murray SA (2014). Development and evaluation of the supportive and palliative care indicators tool (SPICT): a mixed-methods study. BMJ Support Palliat Care.

[9] Downar J, Goldman R, Pinto R, Englesakis M, Adhikari NK (2017). The “surprise question” for predicting death in seriously ill patients: a systematic review and meta-analysis. CMAJ..

[10] Afshar K, Feichtner A, Boyd K (2018). Systematic development and adjustment of the German version of the supportive and palliative care indicators tool (SPICT-DE). BMC Palliat Care.

[11] De Bock R, Van Den Noortgate N, Piers R (2018). Validation of the supportive and palliative care indicators tool in a geriatric population. J Palliat Med.

[12] Fachado AA, Martínez NS, Roselló MM (2018). Spanish adaptation and validation of the supportive & palliative care indicators tool - SPICT™-ES. Rev Saúde Pública.

[13] Beaton DE, Bombardier C, Guillemin F (2000). Guidelines for the process of cross-cultural adaptation of self-report measures. Spine.

[14] World Health Organization, et al. Process of translation and adaptation of instruments. https://www.who.int/substance_abuse/research_tools/translation/en/ Accessed 20 Dec 2019.

[15] Polit DF, Beck CT, Owen SV (2007). Is the CVI an acceptable indicator of content validity? Appraisal and recommendations. Res Nurs Health.

[16] Zamanzadeh V, Ghahramanian A, Rassouli M (2015). Design and implementation content validity study: development of an instrument for measuring patient-centered communication. J Caring Sci.

[17] Brennan P, Perola M, Van Ommen GJ (2017). Chronic disease research in Europe and the need for integrated population cohorts. Eur J Epidemiol.

[18] Global Atlas of Palliative Care at the end of life. Link to: https://www.who.int/nmh/Global_Atlas_of_Palliative_Care.pdf Accessed 28 March 2019.

[19] Temel JS, Greer JA, Muzikansky (2010). Early palliative care for patients with metastatic non-small-cell lung cancer. N Engl J Med.

[20] Zimmermann C, Swami N, Krzyzanowska M (2014). Early palliative care for patients with advanced cancer: a cluster-randomised controlled trial. Lancet.

[21] Leysen B, Van den Eynden B, Janssens A (2019). Recruiting general practitioners for palliative care research in primary care: real-life barriers explained. BMC Fam Pract.

[22] Scaccabarozzi G, Amodio E, Pellegrini G (2018). The “ARIANNA” project: an observational study on a model of early identification of patients with palliative care needs through the integration between primary care and Italian home palliative care units. J Palliat Med.

