# Supplementary material for: Supportive and palliative care indicators tool (SPICT™): content validity, feasibility and pre-test of the Italian version
Source: BMC Palliat Care. 2020 Jun 6;19:79. doi: 10.1186/s12904-020-00584-3 (PMC7276086; doi:10.1186/s12904-020-00584-3)
Supplement: Supplementary file 1 — Additional file 1. [file 12904_2020_584_MOESM1_ESM.pdf]

THE UNIVERSITY *of* EDINBURGH

Supportive and Palliative Care Indicators Tool (SPICT™)

The SPICT™ is used to help identify people whose health is deteriorating. Assess them for unmet supportive and palliative care needs. Plan care.

Look for any general indicators of poor or deteriorating health.

- Unplanned hospital admission(s).
- Performance status is poor or deteriorating, with limited reversibility. (eg. The person stays in bed or in a chair for more than half the day.)
- Depends on others for care due to increasing physical and/or mental health problems.
- The person's carer needs more help and support.
- The person has had significant weight loss over the last few months, or remains underweight.
- Persistent symptoms despite optimal treatment of underlying condition(s).
- The person (or family) asks for palliative care; chooses to reduce, stop or not have treatment; or wishes to focus on quality of life.

Look for clinical indicators of one or multiple life-limiting conditions.

Cancer

Functional ability deteriorating due to progressive cancer.

Too frail for cancer treatment or treatment is for symptom control.

Dementia/ frailty

Unable to dress, walk or eat without help.

Eating and drinking less; difficulty with swallowing.

Urinary and faecal incontinence.

Not able to communicate by speaking; little social interaction.

Frequent falls; fractured femur.

Recurrent febrile episodes or infections; aspiration pneumonia.

Neurological disease

Progressive deterioration in physical and/or cognitive function despite optimal therapy.

Speech problems with increasing difficulty communicating and/or progressive difficulty with swallowing.

Recurrent aspiration pneumonia; breathless or respiratory failure.

Persistent paralysis after stroke with significant loss of function and ongoing disability.

Heart/ vascular disease

Heart failure or extensive, untreatable coronary artery disease; with breathlessness or chest pain at rest or on minimal effort.

Severe, inoperable peripheral vascular disease.

Respiratory disease

Severe, chronic lung disease; with breathlessness at rest or on minimal effort between exacerbations.

Persistent hypoxia needing long term oxygen therapy.

Has needed ventilation for respiratory failure or ventilation is contraindicated.

Kidney disease

Stage 4 or 5 chronic kidney disease (eGFR < 30ml/min) with deteriorating health.

Kidney failure complicating other life limiting conditions or treatments.

Stopping or not starting dialysis.

Liver disease

Cirrhosis with one or more complications in the past year:

- diuretic resistant ascites
- hepatic encephalopathy
- hepatorenal syndrome
- bacterial peritonitis
- recurrent variceal bleeds

Liver transplant is not possible.

Other conditions

Deteriorating and at risk of dying with other conditions or complications that are not reversible; any treatment available will have a poor outcome.

Review current care and care planning.

- Review current treatment and medication to ensure the person receives optimal care; minimise polypharmacy.
- Consider referral for specialist assessment if symptoms or problems are complex and difficult to manage.
- Agree a current and future care plan with the person and their family. Support family carers.
- Plan ahead early if loss of decision-making capacity is likely.
- Record, communicate and coordinate the care plan.

Please register on the SPICT website (www.spict.org.uk) for information and updates

SPICT™, April 2017
